# Biomaterials-Based Antioxidant Strategies for the Treatment of Oxidative Stress Diseases

**DOI:** 10.3390/biomimetics9010023

**Published:** 2024-01-03

**Authors:** Maria Perez-Araluce, Tomasz Jüngst, Carmen Sanmartin, Felipe Prosper, Daniel Plano, Manuel M. Mazo

**Affiliations:** 1 Biomedical Engineering Program, Enabling Technologies Division, CIMA Universidad de Navarra, 31008 Pamplona, Spain; mparaluce@unav.es; 2 Department for Functional Materials in Medicine and Dentistry, Institute of Functional Materials and Biofabrication, University of Würzburg, D-97070 Würzburg, Germany; 3 Bavarian Polymer Institute, University of Bayreuth, 95447 Bayreuth, Germany; 4 Department of Pharmaceutical Science, Universidad de Navarra, 31008 Pamplona, Spain; sanmartin@unav.es; 5 Hematology and Cell Therapy Area, Clínica Universidad de Navarra and Instituto de Investigación Sanitaria de Navarra (IdiSNA), 31008 Pamplona, Spain; fprosper@unav.es; 6 Centro de Investigacion Biomedica en Red de Cancer (CIBERONC) CB16/12/00489, 28029 Madrid, Spain; 7 Hemato-Oncology Program, Cancer Division, CIMA Universidad de Navarra, 31008 Pamplona, Spain

**Keywords:** biomaterial, antioxidant, oxidative stress, wound healing, neurodegenerative diseases, cardiovascular disease, osteoporosis, tissue engineering

## Abstract

Oxidative stress is characterized by an increase in reactive oxygen species or a decrease in antioxidants in the body. This imbalance leads to detrimental effects, including inflammation and multiple chronic diseases, ranging from impaired wound healing to highly impacting pathologies in the neural and cardiovascular systems, or the bone, amongst others. However, supplying compounds with antioxidant activity is hampered by their low bioavailability. The development of biomaterials with antioxidant capacity is poised to overcome this roadblock. Moreover, in the treatment of chronic inflammation, material-based strategies would allow the controlled and targeted release of antioxidants into the affected tissue. In this review, we revise the main causes and effects of oxidative stress, and survey antioxidant biomaterials used for the treatment of chronic wounds, neurodegenerative diseases, cardiovascular diseases (focusing on cardiac infarction, myocardial ischemia-reperfusion injury and atherosclerosis) and osteoporosis. We anticipate that these developments will lead to the emergence of new technologies for tissue engineering, control of oxidative stress and prevention of diseases associated with oxidative stress.

## 1. Introduction

### What Is Oxidative Stress?

The importance of oxidative stress and its effect on human health is becoming increasingly evident. An in-depth study of how it is generated, its mechanisms and consequences can be very useful for the treatment of numerous diseases. Progress in this field has led to the development of numerous strategies to tackle it, including the development of antioxidant materials as well as the development of new strategies for the effective delivery of antioxidants. This work aims to comprehensively show these biomaterial-based antioxidant strategies focusing on the treatment of chronic wounds, neurodegenerative, cardiovascular and bone diseases.

In chemistry, a free radical is a chemical species with one or more unpaired electrons. It can react with other molecules by donating its unpaired electron to another molecule or by accepting an electron from another molecule to become more stable. This reaction results in the formation of a new free radical and, thus, in a chain reaction that propagates until quenching [1]. Free radicals are formed physiologically. In the mitochondria, an oxygen molecule can be reduced to water because of the production of adenosine triphosphate (ATP) during the respiration process. The intermediate steps of oxygen reduction involve the formation of various free radicals or reactive oxygen species (ROS): the superoxide anion radical (O_2_-), hydrogen peroxide (H_2_O_2_) and the hydroxyl radical (OH) [2]. The formation of the superoxide anion radical stems from the reduction of oxygen by one electron, hydrogen peroxide by two electrons and the hydroxyl radical by three electrons. Furthermore, molecular oxygen can undergo electronic excitation to form singlet molecular oxygen (^1^O_2_). Additionally, various cellular sources contribute to ROS production, arising from different enzymes such as nitric oxide synthase, nicotinamide adenine dinucleotide phosphate (NADPH) oxidases (NOXs) and xanthine oxidase in mitochondria, as well as from peroxisomal constituents [3]. During protein folding and disulphide bond formation within the endoplasmic reticulum, oxidants are released [1]. Oxygen radicals can occur as alkyl or peroxyl radicals, as they can react with susceptible compounds, including lipids, proteins and/or DNA. Physiologically, the rate of quenching of free radicals is greater than that of the reactions between free radicals and other cellular components, so the basal concentration of free radicals is kept low [4].

ROS have emerged as versatile signalling molecules that orchestrate a wide spectrum of physiological processes. For instance, they play a crucial role in activating hypoxia-inducible factors (HIFs) [5], which, in turn, induce the expression of erythropoietin (EPO) to boost red blood cell production, vascular endothelial growth factor (VEGF) to stimulate angiogenesis and glycolytic enzymes to maintain ATP levels under hypoxic conditions [6]. On the other side, increased ROS in the body can induce autophagy through a variety of signalling pathways [7,8]. At low levels, increased ROS also activates the immune response [9,10]. The importance of ROS in the immune response is demonstrated by the fact that individuals with an inherited deficiency in some of the enzymes that produce physiological ROS develop chronic granulomatous disease (CGD) and are unable to defend themselves against common infections [11].

**Figure 1:**
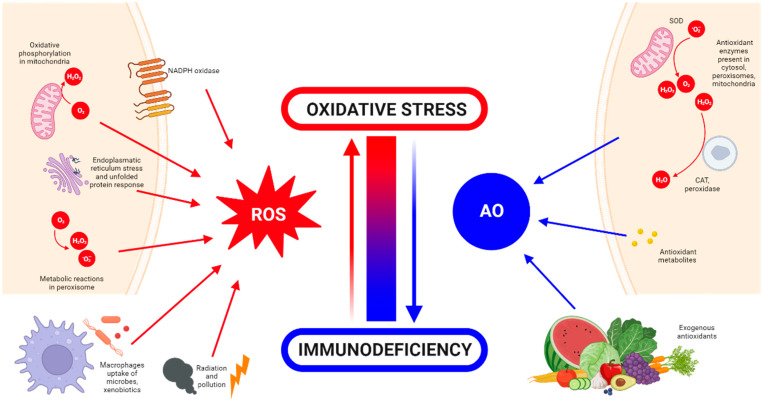
ROS and antioxidant (AO) generation and sources in the body. Made in ©BioRender-biorender.com.

The body has antioxidant mechanisms to quench excessive ROS production [12]. Antioxidants can be classified into endogenous and exogenous ones. Endogenous antioxidants are generated by the body itself. They can be enzymatic, such as superoxide dismutase (SOD), catalase, glutathione peroxidase and glutathione reductase, or non-enzymatic, such as some metabolites (including lipoid acid, glutathione, *L*-arginine, melatonin and bilirubin) [13]. Exogenous antioxidants are supplied externally through food and drink intake, as these nutrients cannot be produced by the body and must be incorporated through the diet. They include molecules such as vitamins E and C, carotenoids, trace metals (selenium, zinc and manganese), flavonoids, omega 3 and 6 fatty acids, etc. [14]. The sources and generation of ROS and antioxidants are summarized and illustrated in Figure 1.

Oxidative stress, first proposed in 1990 by Sohal and Allen [15], is defined as a disturbance in the balance between oxidant production and antioxidant activity [16]. This imbalance can arise either from excessive ROS generation or from a decline in the scavenging capacity or availability of antioxidants. The accumulation of ROS due to this imbalance leads to oxidative damage, affecting essential cellular macromolecules such as DNA, proteins and lipids. Over time, this damage can contribute to the development of chronic inflammation [17]. Inflammation has long been recognized as a primary driver of ROS overproduction, a physiological response triggered by detrimental stimuli and conditions such as infection or tissue injury. This response is aimed at neutralization of the damaging agent and homeostatic restoration of the tissues involved. Due to the complexity of the inflammatory response, it requires careful regulation to initiate, maintain, aggravate or modulate the inflammatory response. A restrained inflammatory response is generally considered beneficial but can be destructive when dysregulated and chronic. Chronic inflammation has been implicated in the pathogenesis of several diseases, including cancer, cardiovascular diseases and osteoarthritis, among others [18,19]. When the body is stimulated by exogenous and proinflammatory factors such as the exposure to excessive sunlight, pathogens or chemicals, ROS generated within the body can surpass the antioxidant defence capacity of cells, leading to a disruption in redox homeostasis [7,20].

## 2. Detrimental Effects of ROS

Oxidative stress has been demonstrated to play a key role in the pathobiology of multiple organs, including the lungs, brain, skin, joints, bones, kidneys, eyes, heart and blood vessels. Increased ROS is also related to multiorgan diseases such as cancer, aging, diabetes, inflammation and infections [18]. These diseases align with the consequences of elevated ROS levels within cells. As previously mentioned, ROS can inflict detrimental effects, including DNA or RNA damage [21], membrane lipid and phospholipid peroxidation, and protein oxidation [22]. This damage is often irreversible and prevents cells from performing their physiological functions. 

**Figure 2:**
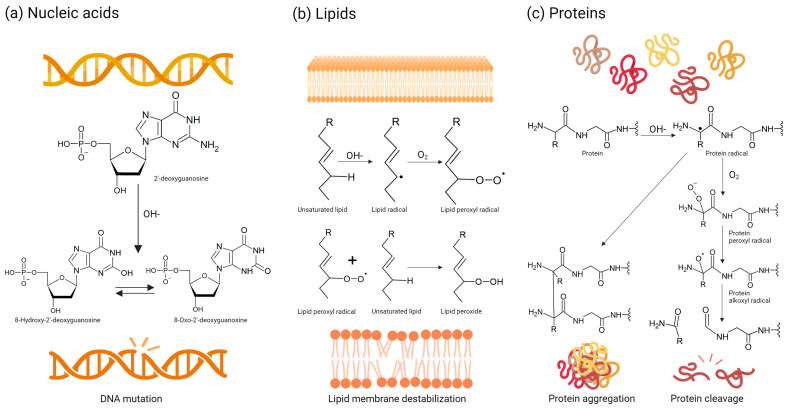
Main reactions between ROS and biomolecules, and detrimental effects of ROS in the body. (**a**) DNA alterations by ROS. (**b**) Lipid peroxidation by ROS. (**c**) Protein modifications by ROS. Made in ©BioRender-biorender.com.

Amongst nucleic acids, mitochondrial DNA is often more susceptible to oxidative damage caused by ROS generated by the respiratory chain, largely owing to its proximity [21]. Mitochondrial DNA mutations have been linked to various disorders, particularly neurodegenerative diseases [23]. Nuclear DNA and RNA can also be targeted by ROS. The primary mechanism of ROS-induced DNA damage involves the reaction between the hydroxyl radical and the double bonds of DNA’s nitrogenous bases, resulting in hydroxylated adduct formation. The most studied bioproduct of this reaction is 8-oxoguanine or 8-hydroxyguanine from guanine [24]. The hydroxyl adducts generated by hydroxyl radical attack can themselves react further with DNA, initiating a cascade of oxidative damage and leading to the formation of additional ROS. The hydroxyl radical, in addition to reacting with the nitrogenous bases, can also react with sugar by subtracting oxygen from the 2′-deoxyribose residues causing strand breaks (Figure 2a) [25]. These types of alterations have been linked to some diseases such as cancer [26]. Furthermore, with these types of modifications, ROS can also attack nucleosomes leading to DNA unpacking and fragmentation. These genetic and epigenetic alterations can result in the deregulation of oncogenes and tumour suppressor genes [27,28].

The unsaturated fatty acids in cell membranes can be oxidized by ROS, resulting in the formation of lipid peroxides. Elevated levels of lipid peroxides are linked to atherosclerosis, heart failure, Alzheimer’s disease, rheumatoid arthritis, cancer and various immunological disorders [29]. Free radicals that come into contact with lipids initiate oxidation of the lipid chains, resulting in the formation of hydroperoxidized lipids and alkyl radicals. In the context of cell membranes, this effect is particularly significant due to the substantial presence of lipids. The alkyl radicals formed during the process are extremely reactive and can react with other polyunsaturated fatty acids, causing the chain reaction to continue until two free radicals react with each other. The structure and physical properties of the membrane are altered by these chemical changes, affecting its integrity, fluidity and, in turn, function (Figure 2b) [30].

Proteins are also affected by oxidative stress. Both non-enzymatic and enzymatic proteins are indispensable for the structural integrity and functional processes of the body. ROS can lead to dysregulation of enzyme catalytic activity and metabolic pathways. Proteins are made up of amino acids, which can be oxidized by ROS. The peptide bond that joins amino acids can be affected by ROS, resulting in its cleavage. Finally, oxidative stress can also cause protein aggregation (Figure 2c). Depending on the affected tissue, these effects have been linked to neurodegenerative diseases, rheumatoid arthritis and other diseases [31].

Dietary supplementation of antioxidants has been attempted to reduce the effects of inflammation and reduce oxidative stress, but their low bioavailability and stability are major drawbacks. Indeed, in the same way as ROS, antioxidants are highly reactive, making storage difficult due to their rapid oxidation. Similarly, their reactivity, even after in vivo administration, can lead to low bioavailability [32]. To improve the bioavailability of potential antioxidant compounds, materials and biomaterials have been devised, particularly in the domain of tissue engineering, to facilitate sustained and prolonged exposure of antioxidant compounds.

## 3. Oxidative Stress and Tissue Engineering

Tissue engineering (TE) aims at reproducing as accurately as possible the properties and functionality of the concerned tissue. For this, TE relies on the interdisciplinary blending of material science, biomedicine and engineering disciplines, amongst others. Its applications not only include regenerative medicine, but also disease modelling, drug testing and personalized medicine amongst others [33,34,35]. However, to reproduce the natural composition of a tissue, it is crucial to duplicate not only the cellular components but also the extracellular matrix, encompassing their spatial arrangement within the 3D environment.

Various materials have been utilized to mimic the extracellular matrix (ECM) of tissues. One of the most significant categorizations is based on the physical consistency of the employed material. This distinction delineates hydrogels, polymeric scaffolds and composite systems. Hydrogels are commonly highly hydrophilic, three-dimensional networks capable of absorbing substantial quantities of water or biological fluids. They are arguably the most extensively investigated material. Among them, natural polymers like collagen and gelatine (denatured/digested collagen) have been extensively used to produce diverse semi-synthetic materials [36], such as gelatine methacryloyl (GelMA) [37]. Other materials have been used to form hydrogels, from chitosan or hyaluronic acid to alginate and beyond. Of the many natural hydrogels, decellularized extracellular matrix (dECM) has been a subject of considerable interest since the groundbreaking discovery of the process, particularly in the context of tissue engineering [38,39]. In theory, dECM preserves the entire biochemical makeup of the ECM of origin, establishing a more intricate and biomimetic environment. Synthetic hydrogels, such as polyethylene glycol (PEG), polyglycolic acid (PGA) and polylactic-glycolic acid (PLGA), have also been extensively investigated, but they often raise concerns regarding biocompatibility or cellular adhesion [40]. Polymeric scaffold structures are being increasingly used in recent years, as they can supply a different range of characteristics to those of hydrogels, namely control over spatial composition and microstructure, and also excellent mechanical properties, which can be custom-tailored according to a specific tissue. In addition, implementation of fabrication technologies such as additive manufacturing, can result in scaffolds with highly controlled structures (reviewed in [41]). Polymers that can be processed into fibres can also be classified according to their origin. Examples of natural polymers are collagen, keratin, elastin, silk, chitosan, cellulose and hyaluronic acid. Among the synthetic polymers, the most commonly used are poly-ε-caprolactone (PCL) [42,43], poly(lactic acid) (PLA), poly(lactic-co-glycolic acid) (PLGA), poly-(glycerol-sebacate) (PGS) [44] and poly(glycolic acid) (PGA) [45]. Finally, composites generated using hydrogels and polymeric scaffolds can provide the best of both worlds. For example, one of the general drawbacks of hydrogels is their low mechanical properties. However, polymeric scaffolds display wide fabrication plasticity, as they can be processed by several methods, with the same material having different properties and possibilities depending on the fabrication technology used [46]. Blending of these components can deliver composite systems that feature different micro- and macro-mechanical properties. Currently, the most employed manufacturing modalities are mould-casting, 3D build and seed technologies, and bioprinting, but there are others such as cryogelation, gas foaming, thermally induced phase separation, solution electrospinning and textile-based fabrication (reviewed in [41]).

However, TE is not only about replacing damaged tissue, but also about using engineering knowledge to repair it. This is why biomaterials with therapeutic effects are being developed. In this way, TE becomes not only a replacement option but also a curative one. This strategy is particularly interesting when the aim of the therapy is a controlled release of the drug or when an in situ release of the drug is necessary to avoid its possible adverse effects on other organs, or to improve its bioavailability. In the case of oxidative stress, it is a very interesting avenue, since one of the main disadvantages of treatment with antioxidants is their low bioavailability. In addition, when dealing with chronic inflammation, materials-based strategies would permit the controlled release of antioxidants in the tissue. It can also be beneficial for cell repopulation and tissue regeneration after transplantation [47], as the injured site is often accompanied by oxidative stress and inflammation that was already present. It is a general issue that during cell culture ROS are generated, and they trigger oxidative stress that is detrimental to cells in a biomaterial scaffold and for its prospective transplantation. In addition, potential injury during the procedure can alter the physiological oxygen levels of the tissue. Taken together, these conditions may increase inflammation and decrease the chances for a successful TE approach [48]. Therefore, ROS-scavenging materials can be of great use in this area.

**Figure 3:**
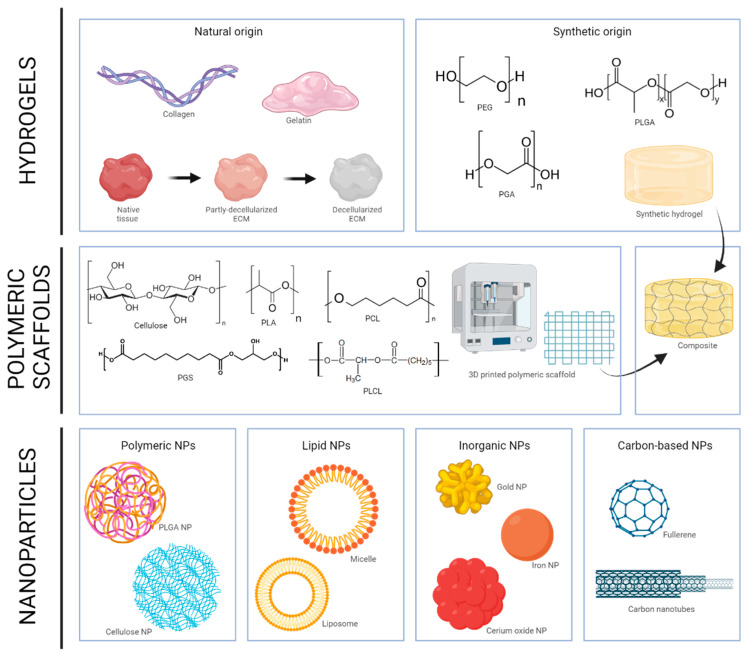
Biomaterials employed in tissue engineering (TE). Made in ©BioRender-biorender.com.

In addition to the biomaterials mentioned above (hydrogels, polymeric scaffolds and composites), traditional “hard and stiff” biomaterials, and materials in the form of nanoparticles (NPs) have also been developed. Traditional materials such as titanium alloys and bioceramics have been modified with anti-inflammatory coatings to add a therapeutical advantage [49]. In the case of NPs, their versatility lies in their ability to accommodate functionality, allowing molecules to be encapsulated within or coupled to their surfaces. NPs loaded with antioxidants have been extensively investigated for the mitigation of oxidative stress. The diverse types of NPs can be classified based on the employed material, encompassing carbon-based, lipid-based, inorganic and polymeric NPs [50]. All these biomaterials employed in TE are summarized in Figure 3.

Amid all the pathologic conditions that can be the target of TE strategies with antioxidant materials, wound healing disorders, neurodegenerative, cardiovascular and bone diseases are of special interest due to their high impact. In the following sections, we revise published research on applying TE strategies with antioxidant materials in these diseases.

## 4. Biomaterials Employed for Oxidative Stress Diseases

### 4.1. Wound Healing

A wound is a breach or disruption in the skin’s integrity arising from physical or thermal injury. Depending on the area of skin affected, there may be a series of alterations in the organism such as blood loss, dehydration, difficulty in maintaining body temperature or infections [51]. The wound healing process encompasses an intricate sequence of biological events aimed at reconstituting the skin barrier function. Four distinct stages characterize the normal wound healing process [52]: homeostasis, inflammation, proliferation and remodelling. The homeostasis stage, initiated immediately post-injury, entails the accumulation of a fibrin-rich fluid that promotes clotting and wound stabilization. Then, the inflammatory stage ensues, characterized by the influx of inflammatory cells, including leukocytes, monocytes and macrophages, which orchestrate the removal of cellular debris, stimulating cell migration, angiogenesis and tissue remodelling. Afterwards, the proliferation stage commences, featuring the vigorous regrowth of epithelial cells and fibroblasts, effectively reconstructing the damaged tissue. Ultimately, the remodelling stage commences, culminating in the formation of stable scar tissue, comprising connective tissue and a newly established epithelium [53]. However, disruption of these factors and stages can lead to chronic wound healing. 

Extensive research has demonstrated that moderate levels of ROS facilitate healthy wound healing by stimulating cell migration and angiogenesis, whereas excessive ROS can contribute to chronic wounds [53,54,55]. In chronic wounds, a persistent inflammatory response triggers a substantial buildup of ROS, surpassing the body’s natural antioxidant defences, hindering cell migration and proliferation, effectively blocking the transition from the inflammatory stage to the proliferative phase and subsequent tissue remodelling [56]. Furthermore, ROS and pro-inflammatory cytokines can stimulate the excessive production of serine proteases and matrix metalloproteinases, leading to the degradation of the ECM, which in turn exacerbates infection and hinders tissue repair and wound healing [57]. This vicious cycle of prolonged inflammation perpetuates chronic wound healing, characterized by the absence of healing after six weeks [58]. Research has found that antioxidants can promote wound healing, particularly in the case of chronic wounds. Hence, the addition of an antioxidant component to the engineered tissue appears to be one of the most promising current therapies [59].

**Figure 4:**
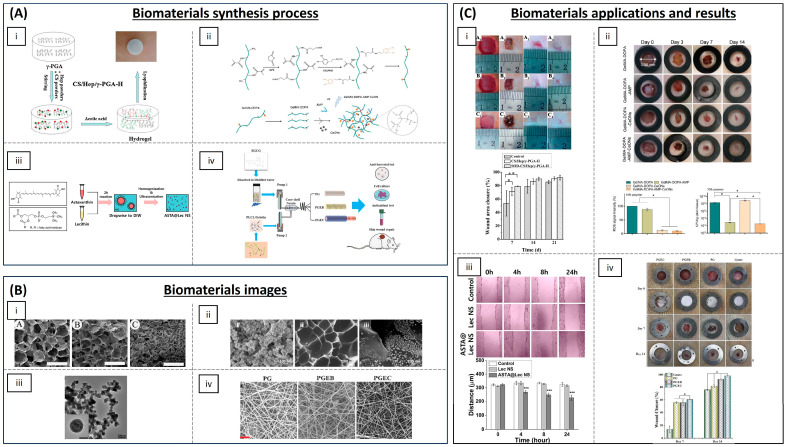
Salient biomaterials currently used for the treatment of chronic wounds. (**A**) Synthesis process of the biomaterials. (**i**) Preparation of CS/Hep/γ-PGA composite hydrogels [61]. (**ii**) Synthesis reaction for the preparation of CeON-loaded GelMA-DOPA hydrogel [65]. (**iii**) Development of astaxanthin-loaded lecithin nano-liposol (ASTA@Lec NS) [77]. (**iv**) Preparation of electrospun PLCL/gelatine nanofibre membranes (NM) (PG, PGEB and PGEC) [81]. (**B**) Biomaterials images. (**i**) SEM images of CS/Hep/γ-PGA hydrogels with different ratios of synthesis: A: 10/1/9, B: 10/3/7 and C: 10/5/5 (from left to right) (scale bar: 1 mm) [61]. (**ii**) SEM observation of CeONs (left (**i**)) and CeON-loaded GelMA-DOPA hydrogel (middle (**ii**) and right (**iii**)) [65]. (**iii**) Transmission electron microscopy (TEM) image of ASTA@Lec NS (scale bar: 200 nm) [77]. (**iv**) SEM image of the different nanofibre membranes synthesized (scale bar: 8 μm) [81]. (**C**) Wound healing results of the biomaterials. (**i**) Zhang L. et al. showed that the wounds treated by SOD-CS/Hep/γ-PGA-H (middle) showed a better closure ratio of 92.0% ± 3.7% versus those of the control group (top) (85.4% ± 2.4%) and the same hydrogel without SOD (bottom) (89.8% ± 2.8%) at days 7, 14 and 21 (left to right). The graph shows the wound closure rate (n = 3). * *p* < 0.05; ** *p* < 0.01 [61]. (**ii**) Cheng H et al. determined the healing speeds of the skin defects treated with different GelMA-DOPA-based hydrogels. It was found that GelMA-DOPA-AMP-CeONs facilitated the most rapid healing in the defect areas, followed by the GelMA-DOPA-AMP and GelMA-DOPA-CeONs groups. The left graph shows a decreased in vivo ROS detection of the wound covered with GelMA-DOPA-based hydrogels at day 2 than controls. The right graph demonstrated the antimicrobial effect of the synthesized hydrogel. * *p* < 0.05 [65]. (**iii**) Oh H et al. showed that the scratch distance of the ASTA@Lec NS-treated cells decreased from 324 ± 6 μm to 227 ± 19 μm after 24 h of treatment; however, scratch distance of the control group and the group without ASTA exhibited almost no change from that of the initial distance. *** *p* < 0.001 [77]. (**iv**) Li A et al. demonstrated that the highest wound closure rate was observed in the group treated with PGEC (98%), whereas the gauze treated group showed the least wound closure rate. * *p* < 0.05 [81]. All images reproduced with permission. Images (**A**(**i**),**B**(**i**),**C**(**i**)) [61] 2012, Elsevier. Images (**A**(**ii**),**B**(**ii**),**C**(**ii**)) [65] 2021, Elsevier. Images (**A**(**iii**),**B**(**iii**),**C**(**iii**)) [77] 2020, Oh H et al. Images (**A**(**iv**),**B**(**iv**),**C**(**iv**)) [81] 2022, Elsevier.

Antioxidant materials for the treatment of chronic wounds are summarized in Table 1 and some of them are visualized in Figure 4. Hydrogels are a widely used type of material for the treatment of chronic wounds as they are air-permeable and have the ability to absorb wound exudate. Being aqueous, they also retain humidity, allowing bioactive agents to be loaded. In addition, they can reduce the surface temperature of the wound, which can help to alleviate the patient’s pain [60]. Hydrogels loading antioxidant molecules or particles are being assayed for the treatment of chronic wounds. Recent research suggests that superoxide dismutase (SOD)-loaded hydrogels exhibit efficacy in mitigating ROS generation and oxidative stress associated with chronic wounds. Zhang et al. have developed hydrogels composed of varying proportions of chitosan, heparin and poly(γ-glutamic acid). Their findings demonstrate that this formulation can expedite wound healing in diabetic rats by accelerating re-epithelialization and collagen deposition [61]. Dong et al. have developed an injectable and thermo-sensible hydrogel-poly(*N*-isopropyl-acrylamide)/poly(γ-glutamic acid) loaded with SOD that could eliminate the superoxide anion. Its use in a diabetic rat model revealed good biocompatibility in vitro and demonstrated a superior wound closure rate compared to control groups [62]. Prussian Blue nanoenzyme is known to possess SOD-, catalase- and peroxidase-mimicking activities through various redox pathways [63]. Sahu et al. have used Prussian Blue NPs and demonstrate its strong superoxide scavenging activity and hydrogen peroxide degradation capacity in addition to deposition, maturation and arrangement of collagen fibres in a cutaneous wound model in mice [64]. Other hydrogels loading antioxidant NPs have also been developed, such as the one published by Chen et al. They formulated a sprayable hydrogel based on methacryloyl gelatine (GelMA), a photopolymerizable gelatine derivative, with dopamine motifs for wound dressing loaded with cerium oxide NPs (CeONPs) and the antimicrobial peptide (AMP) HHC-36 8 (KRWWKWWRR), a cationic peptide with broad spectrum antibacterial effect, seeking combined ROS-scavenging and antibacterial properties. When administered to rats, these novel hydrogels exhibited effective skin restoration at the treatment site even in the presence of severe infection and inflammation [65]. CeONPs have also been used combined with microRNA-146, as it is getting dysregulated in diabetic wounds, loaded into a zwitterionic cryogel (gels formed below freezing temperatures). The hydrogel was composed of [2-(methacryloloxy)ethyl]dimethyl-(3-sulphopropyl) ammonium hydroxide (SBMA) and 3-[[2-(methacryloyloxy)ethyl] dimethylammonio] propionate (CBMA) and crosslinked with 2-hydroxyethyl methacrylate (HEMA) which led to the development of a multifunctional system that can be applied topically and injected, and heals itself, ensuring controlled and prolonged release of therapeutic molecules. The hydrogel was tested in mice and demonstrated that it could accelerate diabetic wound healing [66]. Silver NPs have been encapsulated in hydrogels. Silver is known to possess antibacterial properties and its anti-inflammatory properties have been demonstrated as it enhances apoptosis in inflammatory cells while decreasing pro-inflammatory interleukin levels [67]. Masood et al. have developed a silver NP impregnated chitosan–polyethylene glycol (PEG) hydrogel which showed enhanced antioxidant activity and wound healing capacity in diabetic rabbits [68]. Chitosan has also been reported to display antioxidant properties [69]. Nanochitosan NPs produced by gamma irradiation have been encapsulated in a bacterial cellulose polymer matrix, showing antioxidant and antimicrobial activity in vitro [70]. Polyphenols have also been employed to enhance the antioxidant properties of biomaterials. Eugenol, a natural polyphenol, and an allylbenzene derivative present in nutmeg and cinnamon, has been incorporated into chitosan, demonstrating that eugenol phenolic groups improved antioxidant activity in comparison with chitosan alone [71]. Zhao et al. have functionalized quaternized chitosan-g-polyaniline and benzaldehyde functional groups with poly(ethylene glycol)-co-poly(glycerol sebacate). This material showed good self-healing capacity, electro-activity, free radical scavenging capacity and biocompatibility, and, when employed in a full-thickness skin defect mouse model in vivo, it showed blood clotting capacity. This resulted in a significantly enhanced wound healing process in comparison with controls [72]. Other multifunctional polysaccharide hydrogels have exhibited promising outcomes for wound management and pain mitigation when incorporated with antioxidant compounds, such as carboxybetaine dextran and sulphobetaine dextran hydrogels. These hydrogels have undergone in vivo testing in a mouse full-thickness wound model, demonstrating self-healing and antifouling properties, remarkable antioxidative activity, and enhanced resistance to bacterial adhesion [73]. To maximize the sequestration of ROS, a nanocomposite hydrogel was constructed from alginate and positively charged Eudragit nanoparticles encapsulating edaravone, an FDA-approved drug for the treatment of acute cerebral infarction due to its hydroxyl radical scavenging ability. This hydrogel was shown to promote wound healing with dosage-responsive effect and to be an efficient free radical scavenger in a skin-wounded mouse [74]. Zhao et al. have generated a ROS scavenging hydrogel by using polyvinyl alcohol crosslinked by *N*1 -(4-boronobenzyl)-*N*3 -(4-boronophenyl)-*N*1, *N*1, *N*3, *N*3-tetramethylpropane-1, 3-diaminium (a ROS responsive linker) and was incorporated with mupirocin, a broad-spectrum antibiotic, and GM-CSF, a pleiotropic cytokine that facilitates tissue regeneration. The hydrogel could release therapeutics to accelerate wound closure and eliminate bacterial infections in response to endogenous ROS in the wound microenvironment [75]. The combination of different anti-inflammatory compounds such as melanin and berberine was used to form hydrogels with silk fibroin by Maity et al. The hydrogel functioned as a scaffold for tissue re-epithelialization and enhanced wound repair in a diabetic rat model [76].

Research in the field has not been limited to hydrogels but also encompasses liposomal particles and other types of scaffolds. For example, lecithin nano-liposol particles have been developed as a novel carrier of the carotenoid astaxanthin, a strong antioxidant, using a simple emulsion evaporation method. The improved aqueous solubility of the carotenoids led to enhanced stability and efficacy, resulting in superior ROS scavenging and antioxidant capacity in vitro [77]. Fibrous scaffolds can improve the bioavailability of some antioxidant molecules. Porous poly(*L*-lactic acid) (PLA) electrospun fibrous scaffolds have been employed with embedded asiatic acid (2α,23-Dihydroxyursolic acid) as this molecule has demonstrated a significant antioxidant and anti-inflammatory efficiency in a diabetic mouse model [78]. Results showed that asiatic acid can be continuously released and, by alleviating the high oxidative stress, inflammation and infection present in the wound microenvironment, the hydrogel accelerated re-epithelialization and facilitated angiogenesis and ECM formation [79]. Epigallocatechin-3-*O*-gallate (EGCG) has a significantly strong antioxidant activity [80] and Li et al. have overcome the challenge of low EGCG availability in vivo by developing a cost-effective and straightforward wound dressing comprising poly(L-lactic-co-caprolactone) (PLCL)/gelatine/EGCG/core-shell nanofibrous membrane (PGEC) with sustained drug release capability through coaxial electrospinning technology. It promoted wound healing, cellular differentiation and tissue organization in a rat liver trauma model [81].

All in all, many different biomaterial formulations with antioxidant functions have emerged that have been shown to accelerate wound healing. This novel strategy is especially promising in the treatment of chronic wounds, which often lack optimal therapeutic options. These materials are expected to be of great importance for human health. As we have seen, in addition to having antioxidant properties, it is also essential for materials to have potential antimicrobial capacity. Infection leads to increased inflammation and thus oxidative stress, making healing even more difficult. On the other hand, it is also one of its main complications as it can lead to systemic infection. This is why biomaterials with both characteristics can be considered more therapeutically relevant and we expect it to lead to translation to the bedside. Hydrogels are the most developed type of biomaterial, due to their very nature, which includes added capacities that are especially beneficial for the treatment of chronic wounds, such as their capability to effectively absorb exudate, sustain a moist wound environment and provide cooling effects to the wound surface, thereby alleviating the patient’s discomfort.

**Table 1:** Antioxidant materials employed for wound healing diseases.

Category	Material	Load	Model	Properties	Ref.
Hydrogels	Chitosan, heparin and poly(γ-glutamic acid)	SOD	Diabetic rat model	Accelerating re-epithelialization and collagen deposition	[61]
	Poly(N-isopropyl-acrylamide)/poly(γ-glutamic acid)	SOD	Diabetic rat model	Antioxidant activity and high wound closure rate	[62]
	GelMA with dopamine motifs	Cerium oxide NPs and AMP	Rats	(ROS) scavenging and antibacterial properties	[65]
	SBMA, CBMA and HEMA	Cerium oxide and microRNA-146	Mice	Accelerating wound healing	[66]
	Chitosan-PEG	Silver NPs	Diabetic rabbits	Antioxidant and antibacterial activity	[67]
	Chitosan	Eugenol	-	Antioxidant activity	[71]
	Chitosan-g-polyaniline and benzaldehyde	PEG-co-poly(glycerol sebacate)	Mice	Good self-healing, electro-activity and free radical scavenging capacity	[72]
	Carboxybetaine dextran and sulphobetaine dextran	-	Mice	Self-healing, antioxidative and antifouling properties	[73]
	Alginate	Edudragit NPs and Edavarone	Mice	Wound healing promoting and efficient free radical scavenging	[74]
	Polyvinyl alcohol	Mupirocin and GM-CSF	Diabetic mice	Antibacterial activity and wound closure promoting	[75]
	Silk fibroin	Melanin and berberine	Diabetic rat	Re-epithelialization and wound repair promoting	[76]
Inorganic NPs	Prussian Blue NPs	-	Mice	Antioxidant and collagen deposition	[64]
Liposomal particles	Lecithin nano-liposol	astaxanthin	NIH/3T3 cells	ROS scavenging and antioxidant capacity	[77]
Polymeric matrix	Cellulose	Nanochitosan dust	Human gingival cells	Antioxidant and antimicrobial activity	[70]
	PLA	Asiatic acid	Diabetic mouse model	Accelerating re-epithelization, angiogenesis and ECM formation	[79]
	Poly(L-Lactic-co-caprolactone) (PLCL)	EGCG	Rat liver trauma model	Promoting wound healing and tissue organization	[81]

### 4.2. Neurodegenerative Diseases

Neurological disorders are defined as pathologies affecting the brain, as well as the nerves and spinal cord. Within them, there is a large group of degenerative diseases in which there is a gradual decline in neuronal function and a common symptomatology, characterized by memory and movement disorders, and progressive dementia. Alzheimer’s disease (AD) and Parkinson’s disease (PD) stand out due to their high incidence, their severity, and the corresponding social and economic burden [82]. AD, the leading cause of age-related dementia, is characterized by the extracellular accumulation of proteins forming extracellular amyloid-β plaques and intracellular tau tangles [83]. PD is a progressive neurodegenerative disorder characterized by the gradual degeneration of specific dopaminergic neurons in the substantia nigra pars compacta, resulting in reduced dopaminergic levels within the nigrostriatal dopaminergic pathway [84]. Abnormal alterations in the structural arrangement of specific proteins can trigger their aggregation and subsequent accumulation within nerve cells, leading to neuronal cell death. A prominent neuronal protein that undergoes this pathological process is α-synuclein (α-syn), whose aggregation forms Lewy body filaments, known to impair neuronal function [85].

Several neurodegenerative diseases exhibit common features of abnormal ROS signalling: DNA damage, lipid peroxidation and protein aggregation, as well as mitochondrial dysfunction [86,87]. In addition, these abnormalities have been shown to activate microglia and astrocytes, thereby triggering a pro-inflammatory response and increased ROS generation [88]. Thus, a reciprocal action occurs, as accumulated ROS can also lead to chronic neuroinflammation. On the other hand, oxidative stress and neuroinflammation lead to alterations in synaptic plasticity and cognitive deficits [89]. The use of antioxidant therapy employing scavengers that can restore redox balance in the brain may offer both prophylactic and therapeutic potential, particularly if initiated at early stages of disease progression [90,91]. An important question is whether antioxidant treatment can interfere with physiological cellular functions and whether it is possible to achieve physiological ROS values without lowering them below this threshold.

**Figure 5:**
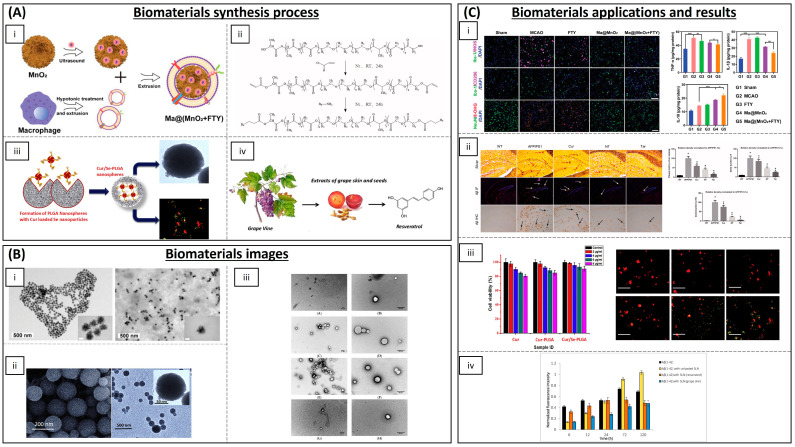
Some biomaterials currently used for the treatment of neurodegenerative diseases. (**A**) Synthesis process of the biomaterials. (**i**) Scheme of the preparation process of Ma@(MnO_2_+FTY) nanoparticles [100]. (**ii**) Synthesis of PLGA-PEG-B6 nanoparticles [104]. (**iii**) Formation of PLGA nanospheres with Cur-loaded selenium NPs [106]. (**iv**) Resveratrol provided from grape skin and grape seed extracts was employed by Loureiro JA et al. to synthesized solid lipid nanoparticles (SLNs) [117]. (**B**) Biomaterials images. (**i**) Representative TEM images of the honeycomb MnO_2_ nanospheres (scale bar: 50 nm) (**left**) and of Ma@(MnO_2_+FTY) nanospheres (scale bar: 50 nm) (**right**) [100]. (**ii**) SEM and TEM images of synthesized nanospheres [104]. (**iii**) TEM images of SLN unloaded (first row), SLN with encapsulated resveratrol (second row), SLN with encapsulated grape skin extract (third row) and SLN with encapsulated grape seed extract (fourth row) (scale bar: 500 nm) [106]. (**C**) Results of the biomaterials as treatments of neurodegenerative diseases. (**i**) Li C et al. demonstrated that the treatment with Ma@(MnO_2_+FTY) in ischemic brains induced a phenotypic transition of microglia from M1 to M2 and reduced oxidative stress (**left**); graphs show that inflammation is also reduced with the treatment with Ma@(MnO_2_+FTY). Scale bar: 100 μm. * *p* < 0.05; ** *p* < 0.01; *** *p* < 0.001 [100]. (**ii**) The study conducted by Fan et al. revealed that Cur-loaded PLGA-PEG-B6 NPs effectively reduced hippocampal Aβ plaques in APP/PS1 mice. Histological analysis demonstrated that APP/PS1 mice exhibited substantial amyloid deposits (indicated by red, white and black arrows) in the hippocampal region. Compared to APP/PS1 control groups, Cur and NT moderately attenuated Aβ plaque formation. Synthetized nanoparticles significantly attenuated this pathology. * *p* < 0.05 versus WT group, # *p* < 0.05 versus former group [104]. (**iii**) Huo X et al. synthesized Cur-loaded Se-PLGA nanospheres; the graph (**left**) shows the good biocompatibility of the nanospheres and non-toxic effects with cell culture. The images (**right**) show the ability of the nanospheres to pass through the BBB and bind to Aβ-amyloid plaques [106]. Scale bar: 100 μm. (**iv**) A decrease in the peptide aggregation was observed when resveratrol and the grape extracts are encapsulated [117]. All images reproduced with permission. Images (**A**(**i**),**B**(**i**),**C**(**i**)) [100] 2021, John Wiley and Sons. Images (**A**(**ii**),**B**(**ii**),**C**(**ii**)) [104] 2018, Taylor & Francis Group. Images (**A**(**iii**),**B**(**iii**),**C**(ii**i**)) [106] 2019, Elsevier. Images (**A**(**iv**),**B**(**iv**),**C**(**iv**)) [117] 2017, MDPI.

One of the most important challenges of therapy in the central nervous system is overcoming the difficulty of entry of materials and drugs into the brain due to the blood–brain barrier (BBB) and the blood–cerebrospinal fluid barrier. The use of tissue engineering strategies is therefore critical, as the use of biomaterials that can cross the BBB will allow more efficient therapy to combat ROS in the brain. The use of nanoparticles (NPs), due to their small size and ability to cross the BBB, has been a main avenue of research for this type of disease; most of them are inorganic NPs, carbon NPs, lipid NPs and polymeric NPs (summarized in Table 2 and some of them are shown in Figure 5).

Inorganic NPs such as CeONPs have been widely explored in neurological diseases. Multiple in vitro studies have demonstrated exceptional promise as antioxidant therapy, suggesting that CeONPs could serve as therapeutic agents to reduce protein aggregation and alleviate the onset of neurodegenerative disorders [92]. Zand et al. performed some in vitro studies that demonstrate that CeONPs reduce the formation of amyloid species and β-sheet structures of α-syn molecules [93]. Siposova et al. studied the bioactivity and anti-amyloid aggregation activity in vitro of CeONPs, using Western blot, fluorescence assays and flow cytometry, showing great benefits in amyloid-related diseases and oxidative stress [94]. It has also been reported that CeONPs exhibit robust anti-ROS activity and, furthermore, have also demonstrated beneficial effects on both neuronal cell differentiation and dopamine production, as demonstrated in studies involving PC12 neuronal cells induced to exhibit a PD-like phenotype [95]. Ceria/Polyoxometalate hybrid NPs (CeONP@POMs) were employed by Guan et al. and their results showed that CeONP@POMs exhibit the ability to promote PC12 cell proliferation, demonstrate efficient BBB penetration and effectively suppress Aβ-induced microglial cell activation, as evidenced by immunofluorescence and flow cytometry analyses [96]. Other metals have also been employed to form antioxidant NPs. Iron oxide NPs (IONPs) are shown to ameliorate neurodegeneration in a Drosophila Alzheimer’s disease model, where IONPs can mimic catalase and can decompose ROS [97]. Yttrium oxide NPs have been employed in vitro with PC12 cells and it was demonstrated that these NPs protect the cells against oxidative stress and apoptosis [98]. The combination of Yttrium NPs and CeONPs was administered intraperitoneally in Wistar rats, showing an improved management of ROS, mediated through the programmed cell death pathway [99]. Manganese dioxide (MnO_2_) nanospheres loaded with fingolimod (an immunomodulating drug) can consume excess H_2_O_2_ and reduce oxidative stress. This reverses microglia from a pro-inflammatory state, by enhancing the survival of damaged neurons in some studies in vitro and in vivo with mice [100].

Carbon nanomaterials have also been employed as antioxidant therapy in neurodegenerative diseases. Rodriguez-Losada et al. have demonstrated in in vitro studies with a mouse-substantia-nigra-derived dopaminergic cell line (SN4741) that partially reduced graphene oxide (PRGO) films prevent dopaminergic neuron loss and α-syn depletion in a molecular environment rendered oxidatively stressed to mimic PD [101]. The protective efficacy of hydrophilic carbon clusters conjugated to poly(ethylene glycol) (PEG-HCCs) was investigated, revealing their ability to shield b.End3 brain endothelial cells and E17 primary cortical neuron cultures from hydrogen-peroxide-induced toxicity [102].

NPs containing antioxidant molecules are also being developed for neurodegenerative applications. Curcumin, resveratrol and epigallocatechin-3-gallate (EGCG) are the most employed. Curcumin (Cur), a yellow pigment derived from the rhizome of *Curcuma longa*, is a prominent constituent of turmeric, a spice widely employed as a colouring agent in food and traditional medical preparations. The beneficial effects of curcumin are based on its strong antioxidant and anti-inflammatory properties [103], but its lipophilic nature and poor bioavailability limit its therapeutic potential. Fan et al. developed a novel brain-targeted NP, poly(lactide-co-glycolide)-block-poly(ethylene glycol) (PLGA-PEG) conjugated with B6 peptide, and loaded it with Cur (PLGA-PEG-B6/Cur). These NPs were administered into HT22 cells and APP/PS1 Al transgenic mice. Their findings demonstrated that PLGA-PEG-B6/Cur effectively improved the spatial learning and memory capability of APP/PS1 mice, along with reducing hippocampal β-amyloid formation and deposit, and tau hyperphosphorylation [104]. Tiwari et al. reported that curcumin-encapsulated poly-lactide-co-glycolic acid (PLGA) NPs (Cur-PLGA-NPs) exhibit a robust capacity to promote neural stem cell (NSC) proliferation and neuronal differentiation in vitro, and in the hippocampus and subventricular zone of adult rats [105]. PLGA NPs loaded with curcumin have been developed by several research groups [106,107,108,109]. Solid lipid Cur NPs (SLCPs) have been demonstrated to enhance the solubility and bioavailability of curcumin. Additionally, studies have indicated that the use of SLCPs produces more potent anti-amyloid, anti-inflammatory and neuroprotective effects than Cur alone [110]. Campisi et al. showed that the encapsulation of curcumin in SLCPs represents an innovative approach for the treatment of AD, as the systemic administration of SLCPs facilitates the modulation of tissue transglutaminase (TG2) isoforms, which play a role in either activating apoptotic pathways or promoting cellular repair in the brains of TgCRND8 mice, an experimental model of AD [111]. NPs combined with metals as iron or gold NPs have also been used for encapsulating curcumin [112,113].

Resveratrol (Res) is a polyphenol naturally occurring in red grapes, peanuts and numerous plant species. It exhibits antioxidant properties, modulates neuroinflammation and promotes adaptive immunity in AD [114]. Res-selenium-peptide NPs act by decreasing Aβ-induced ROS and by enhancing the activity of antioxidant enzymes in PC12 cells [115], as well as downregulating STAT3 expression and interleukin-1β levels, therefore alleviating neuroinflammation in vivo in a rat model of AD [116]. Solid lipid NPs have also been used to load Res. Loureiro et al. functionalized the Res-NPs with the anti-transferrin receptor monoclonal antibody (OX26 mAb) to attain effective brain targeting. Experiments conducted on human brain-like endothelial cells demonstrated highly efficient cellular uptake of OX26-SLNPs and their capacity to effectively inhibit protein aggregation [117]. Other lipid NPs, resveratrol-loaded vitamin E nanoemulsions, have been synthesized for the brain treatment of PD by reducing oxidative stress, achieving higher levels of GSH and SOD in vitro [118].

Epigallocatechin-3-gallate (EGCG) is another polyphenol, the most abundant catechin in tea. Cano et al. incorporated EGCG into PEGylated PLGA NPs with ascorbic acid; they showed that the treatment in mice mitigated neuroinflammation and prevented neuronal loss [119]. Nanolipidic particles containing EGCG significantly enhanced the oral bioavailability of EGCG by over two-fold compared to free EGCG in rats [120]. Other antioxidant molecules, such as quercetin, ferulic acid, ginsenosides, berberine and coenzyme Q10, have also been encapsulated into NPs to increase their bioavailability for the treatment of neurodegenerative diseases [121,122,123,124,125].

The materials described here are shown to be an effective treatment for neurodegenerative diseases by counteracting excessive ROS generation and mostly preventing the aberrant protein aggregation that occurs in the case of AD and PD. The main limitation of biomaterials in the treatment of neuronal diseases is their delivery, so it is essential that they can cross the blood–brain barrier and the blood–cerebrospinal fluid barrier; although some are described as being able to do so, most of them have only been tested in vitro. The size required for a biomaterial to cross the BBB must be less than 200 nm, which makes NPs the best candidates. In addition, their behaviour at the systemic level and their circulation time before being eliminated by the liver or spleen should be studied. On the other hand, more studies would be needed to study their possible long-term neurotoxicity, which also makes clinical trials limited.

**Table 2:** Antioxidant materials employed for neurodegenerative diseases.

Category	Material	Load	Model	Properties	Ref.
Inorganic NPs	Cerium oxide (CeONPs)	-	P12 neuronal cells	Anti-amyloid aggregation, antioxidant activity	[93,94,95]
	Ceria/Polyoxometalates	-	P12 neuronal cells	Inhibition of Aβ-induced microglial cell activation	[96]
	Iron oxide (IONPs)	-	Drosophila Alzheimer’s disease model	Anti-ROS activity	[97]
	Yttrium oxide	-	P12 neuronal cells	Reduction in oxidative stress and apoptosis	[98]
	Yttrium NPs and CeONPs	-	Wistar rats	Reduction in oxidative stress	[99]
	MnO_2_	Fingolimod	Mice	ROS and microglia pro-inflammatory state reduction	[100]
	Selenium NPs	Resveratrol	AD rat model	Anti-inflammatory activity	[116]
Carbon materials	Partially reduced graphene oxide	-	Mouse-substantia-nigra-derived dopaminergic cell line	Prevention of dopaminergic neuron loss and α-syn depletion	[101]
	PEG-HCCs	-	Brain endothelial cell line and primary cortical neuron cells	Protection against hydrogen peroxide	[102]
Polymeric NPs	(PLGA-PEG) and B6 peptide	Curcumin	APP/PS1 Al transgenic mice	Improvement in spatial learning and memory	[104]
	PLGA	Curcumin	Rats	Neuronal differentiation	[105,106,107,108,109]
	PEGylated PLGA NPs	Ascorbic acid and EGCG	Mice	Neuroinflammation and neuronal loss	[119]
Solid lipid NPs	Glycerol behenate	Curcumin	AD mouse model	Cellular damage reduction in brain	[111]
	Cetylpalmitate and OX26 mAb	Resveratrol	Human brain-like endothelial cells	Inhibition of protein aggregation	[117]
	Vitamin E and sefsol	Resveratrol	In vitro	Increasing the levels of GSH and SOD	[118]
	Unspecified	EGCG	Rat	Increasing bioavailability of EGCG	[120]

### 4.3. Cardiovascular Diseases

**Figure 6:**
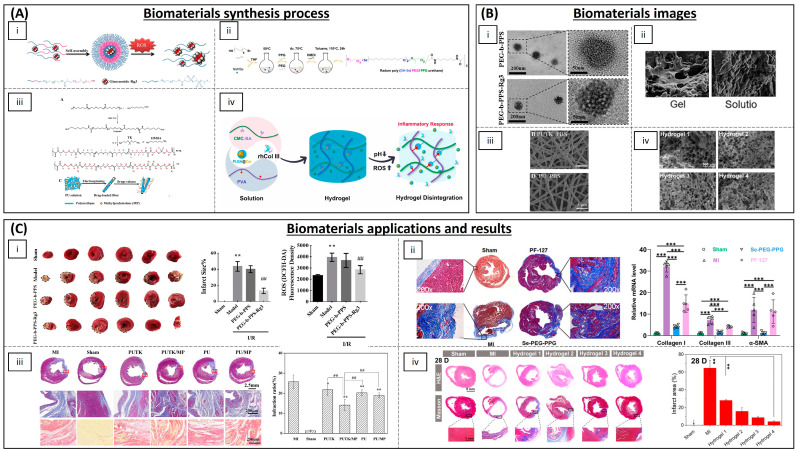
Some biomaterials currently used for the treatment of I/R and cardiac infarct. (**A**) Synthesis process of the biomaterials. (**i**) Self-assembled ROS-responsive polymers of PEG and PPS diblock copolymers were prepared by Li L et al. for the encapsulation of Rg3 [131]. (**ii**) Synthesis pathway of Se-PEG-PPG [164]. (**iii**) Synthesis of polyurethanes (PUTK and PU) that respond to reactive oxygen species (ROS). The polyurethanes were synthesized from poly(ε-caprolactone) diol, 1,6-hexamethylene diisocyanate, and chain extenders of ROS-cleavable thioketal (TK) and 1,6-hexanediamine (HMDA). Additionally, electrospun fibrous patches loaded with methylprednisolone (MP) were fabricated for sustained release [152]. (**iv**) Preparation process and drug release mechanism of MI-responsive hydrogels [161]. (**B**) Biomaterials images. (**i**) TEM images showing the morphology of PEG-b-PPS and PEG-b-PPS-Rg3 (scale bar: 200 nm (**top**); scale bar: 50 nm (**bottom**)) [131]. (**ii**) SEM images of Se-PEG-PPG hydrogel before and after gelation (scale bar: 10 μm) [164]. (**iii**) SEM images of PUTK and PU [152]. (**iv**) SEM images of hydrogels. Hydrogel 1 represented control hydrogel; Hydrogel 2 represented PLGA@Cur NP-encapsulated hydrogel; Hydrogel 3 represented rhCol III-encapsulated hydrogel; Hydrogel 4 represented PLGA@Cur NP and rhCol III-encapsulated hydrogel [161] (**C**) Results of the biomaterials as treatments of neurodegenerative diseases. (**i**) Li L. et al. demonstrated that myocardial infarction area was reduced following intramyocardial injection of PEG-b-PPS-Rg3, whereas the PEG-b-PPS group exhibited no discernible reduction ((**left**) and (**middle**)). Additionally, PEG-b-PPS-Rg3 was found to effectively lower ROS generation (**right**). ** *p* < 0.01 compared with the sham group; ## *p* < 0.01 compared with the model group. [131]. (**ii**) Masson staining results (**left**) indicated that intramyocardial injection with Se-PEG-PPG hydrogel furnished robust structural support for the post-MI heart. Furthermore, they elucidated the inhibitory effects of Se-PEG-PPG hydrogel on the expression of fibrosis-associated genes (**right**). *** *p* < 0.01 [164]. (**iii**) The fibrosis in MI zone decreased significantly after being treated with the fibrous patches synthesized by Yao Y et al. * *p* < 0.05; ** *p* < 0.01 vs MI group. ## represent *p* < 0.01 between the selected groups [152]. (**iv**) Hydrogel 4, composed of PLGA@Cur NP and rhCol III-encapsulated hydrogel, demonstrated remarkable efficacy in improving cardiac function by diminishing infarct size and augmenting the thickness of the left ventricular wall in the infarcted region. ** *p* < 0.01 [161]. Images (**A**(**i**),**B**(**i**),**C**(**i**)) [131] 2020, Elsevier. Images (**A**(**ii**),**B**(**ii**),**C**(**ii**)) [164] 2022, Frontiers. Images (**A**(**iii**),**B**(**iii**),**C**(**iii**)) [152] 2020, Elsevier. Images (**A**(**iv**),**B**(**iv**),**C**(**iv**)) [161] 2022, Elsevier.

Cardiovascular diseases (CVDs) are the leading cause of death worldwide. These include ischemic heart disease contributing to myocardial infarction (MI) and subsequent heart failure (HF) [126]. The pathogenesis of MI is generally attributed to a reduction in, or complete obstruction of, blood flow through the coronary arteries, leading to severe and persistent acute ischemia of the affected myocardium, which in turn causes myocardial cell necrosis. The inflammatory response is initiated in the early stages of MI. While a controlled inflammatory response plays a role in the partial repair of the damaged myocardium, sustained inflammation not only impedes tissue repair but also triggers cardiomyocyte (CM) apoptosis, necrosis and fibrosis, ultimately culminating in ventricular remodelling and cardiac insufficiency [127]. The augmented synthesis and release of proinflammatory cytokines may spread beyond the infarcted area, instigating a second phase of cytokine release that incites interstitial fibrosis and collagen deposition. This inflammation leads to an increase in ROS and oxidative stress, with a reciprocal effect, as in other cases since oxidative stress could also be related to the pathogenesis of these diseases. On the other hand, one of the preferred interventions to restore coronary artery blood supply is reperfusion of the clotted artery by fibrinolytic treatment or coronary stenting. The main drawback of this treatment is that it leads to myocardial ischemia–reperfusion injury (I/R), which also involves an imbalance in ROS. An excess of ROS generated by cardiac tissue inflammation or I/R can activate pro-hypertrophy pathways that stimulate myocardial pathological growth, cellular dysfunction and matrix remodelling. Remodelling occurs progressively, resulting in changes in cardiac size, shape and function [128,129]. Consequently, research endeavours have investigated the application of anti-inflammatory agents to mitigate excessive early inflammation and expedite myocardial repair following MI (Table 3 and Figure 6).

Bae et al. have developed H_2_O_2_-responsive antioxidant copolyoxalates containing vanillyl alcohol (VA) (PVAX), showing that they effectively suppressed the generation of ROS in myocardial ischemia. Their results also demonstrated significantly reduced levels of NADPH oxidase (NOX) 2 and 4 expression, which favours the decrease in ROS generation in the cardiac I/R model in mice [130]. Other polymeric NPs have been developed for I/R antioxidant treatment, most of them based on PEG or PLGA. Copolymers of PEG and poly (propylene sulphide) (PPS) have been used to encapsulate ginsenoside Rg3 (Rg3), a potent antioxidant obtained from Genus panax plants. In a rat I/R model, an intramyocardial injection of Rg3-loaded PEG-b-PPS NPs improved cardiac function and reduced infarct size. Rg3 targets the transcription factor FoxO3a, thereby suppressing the activation of downstream signalling pathways that promote oxidative stress, inflammation and fibrosis [131]. PEG-modified solid lipid NPs carrying baicalin (flavonoid) and schisandrin B (antioxidant and bioactive chemical compounds found in *Schisandra chinensis*) have been developed. They showed heart-targeted drug delivery, enhanced drug penetration and a reduction in the infarct size in rats [132,133]. Exenatide-loaded poly(L-lysine)-poly(ethylene glycol)-poly(L-lysine) (PLL-PEG-PLL) NPs have been also studied and results showed that PLL-PEG-PLL enhance the bioavailability of exenatide, which led to a reduction in oxidative stress injury and an improvement in the myocardial function of I/R injured rats [134].

Different antioxidant molecules have been encapsulated into PLGA NPs for cardiac I/R treatment. Quercetin PLGA NPs were studied in vitro by Lozano et al., demonstrating that the nanoparticles effectively shielded quercetin from degradation and enhanced its bioavailability. Moreover, they exhibited enhanced cell rescue, primarily attributed to a significant reduction in oxidized thiols [135]. Resveratrol has also been employed for heart disease, encapsulated into PLGA NPs, demonstrating that a mitochondria-targeted resveratrol could scavenge the ROS in ischemic CMs and inhibit the apoptosis of damaged CMs [136]. Resveratrol PLGA NPs have also prevented myocardial necrosis and reduced interstitial oedema and neutrophil infiltration in rats [137]. Irbesatan and pioglitazone are approved drugs used for the treatment of diabetes, heart failure and hypertension. In order to enhance their bioavailability in the myocardium and minimize adverse effects, they have been encapsulated in PLGA NPs. It has been shown that they reduced acute inflammation and promoted cardiac repair following MI [138,139]. Ishikita et al. created PLGA NPs loaded with mitochondrial division inhibitor 1 (Mdivi-1). These NPs were able to successfully deliver Mdivi-1 to the cytoplasm and mitochondria of CMs under H_2_O_2_-induced oxidative stress, which simulates I/R injury. In rat neonatal CMs, these NPs showed greater effectiveness in reducing H_2_O_2_-induced cell death than Mdivi-1 alone. Additionally, NP-mediated delivery of Mdivi-1 to the ischemic myocardium protected mice from I/R injury [140]. Coenzyme Q10 (CoQ10), often a food supplement, has been considered as a potential candidate for the treatment of various diseases where oxidative stress plays a significant role, including cardiovascular diseases [141]. Simón-Yarza et al. have encapsulated CoQ10 in PLGA NPs to improve its bioavailability after oral administration and sustained release in mice [142]. Verma et al. administered CoQ10-loaded liposomes to rabbits with experimental MI to enhance intracellular CoQ10 delivery and diminish the proportion of damaged myocardium [143]. On the other hand, activation of the L-type Ca^2+^ channel also contributes to an augmented production and metabolism of mitochondrial ROS [144]. Hardy et al. have studied the concurrent delivery of a peptide targeting the alpha-interacting domain of the L-type Ca2+ channel (AID) in conjunction with the powerful antioxidants curcumin or resveratrol using multifaceted poly(glycidyl methacrylate) (PGMA) nanoparticles. They have shown, in isolated rat hearts subjected to I/R, that this approach can attenuate oxidative stress and superoxide production in cardiac myocytes [145].

Beyond polymeric NPs, metallic and metal–oxide nano-zymes with antioxidant properties have been tested for the treatment of ischemic myocardial damage. Ceria NPs have exhibited protective effects against ROS-induced cell death in vitro, with their ROS-scavenging activity evaluated using SOD and catalase mimetic assays [146]. Cerium oxide NPs exhibit the ability to safeguard cardiac progenitor cells against H_2_O_2_-induced cytotoxicity in a dose- and time-dependent manner in vitro [147]. Gold NPs have been shown to have antioxidant activity [148,149]. Tartuce et al. studied the administration of 2-methoxyisobutylisonitrile-conjugated gold NPs (AuNP-MIBI) in an ischemia–reperfusion model in rats. They showed that inflammation parameters were reduced in animals treated with the NPs but they did not find significant differences in parameters related to oxidative stress [150]. Further studies are needed to confirm its beneficial effect on MI due to its possible side effects [151].

In addition to inorganic NPs, inorganic fibres with antioxidant properties have also been developed. ROS-responsive biodegradable elastomeric polyurethane fibres made by electrospinning containing thioketal linkages and loaded with glucocorticoid methylprednisolone were effectively employed in the treatment of a rat MI model for 28 days. These fibres significantly enhanced cardiac function, including an increase in ejection fraction, a reduction in infarct size and an improvement in myocardial revascularization [152].

Hydrogels with antioxidant properties or loaded with ROS-scavenger molecules have also been widely explored to protect myocardium from oxidative stress after I/R injury. Hao et al. have created a novel injectable hydrogel, constructed from chitosan enhanced with borinate-protected diazeniumdiolate, possessing the capacity to release nitric oxide (NO) (CS-B-NO) in response to ROS stimulation, consequently regulating the ROS/NO imbalance post-I/R. NO, a naturally occurring bioactive gas molecule, plays a pivotal role in safeguarding and regulating cardiovascular function [153]. Administration of CS-B-NO effectively mitigated cardiac damage and detrimental cardiac remodelling, facilitated heart repair and enhanced cardiac function in a mouse model of I/R [154]. Vong et al. developed an injectable hydrogel formed by the electrostatic crosslinking between PMNT-PEG-PMNT triblock copolymer (poly [4-(2,2,6,6-tetramethylpiperidine-*N*-oxyl)aminomethylstyrene]-*b*-poly(ethyleneglycol)-*b*-poly [4-(2,2,6,6 tetramethylpiperidine-*N*-oxyl)aminomethylstyrene) to simultaneously scavenge excessive ROS and modulate local NO expression levels in mice [155]. Polyvinylalcohol/dextran (PVA/Dex) with astaxanthin-loaded hydrogels allowed the in situ release of astaxanthin, a potent natural antioxidant molecule, in the infarcted area and a reduction in the oxidative stress in rats [156]. α-tocopherol liposome loaded chitosan hydrogel was developed by Qu Y. et al. and could suppress the oxidative stress injury in CMs in vitro [157]. Chitosan chloride–glutathione hydrogel in vitro could also effectively scavenge the superoxide anion and the hydroxyl radical [158]. Chitosan–vitamin C (CSVC) hydrogel scaffolds have been shown to enhance CM survival and minimize ROS levels under H_2_O_2_-induced oxidative stress conditions in vitro [159]. Chitosan–gelatine-based hydrogel containing ferulic acid (a potent antioxidant) showed a sustained released of ferulic acid and could protect from oxidative-stress-induced damage in rabbits [160]. Hu et al. have developed an injectable hydrogel composed of phenylboronic-acid-grafted carboxymethyl cellulose (CMC-BA) that exhibits responsiveness to the inflammatory microenvironment at the site of myocardial infarction (MI), delivering curcumin and tailored recombinant humanized collagen type III in a controlled manner, demonstrating remarkable anti-inflammatory properties in rats [161]. Fullerenol NPs have been introduced into alginate hydrogels, effectively scavenging the superoxide anion and hydroxyl radicals in vitro [162]. A hydrogel synthesized from *N*-isopropyl acrylamide and methoxy-PEG methacrylate was injected in vivo into sheep myocardium. Results showed that the hydrogel could improve contractile function, increase wall thickness and decrease ROS after MI [163]. An injectable selenium-containing polymeric hydrogel, namely, poly[di-(1-hydroxylyndecyl) selenide/polypropylene glycol/polyethylene glycol urethane] (poly(DH-SE/PEG/PPG urethane), was synthesized by combining a thermosensitive PPG block, DH-Se (with oxidation-reduction properties) and hydrophilic PEG segments. It was injected in a mouse model of MI and showed an inhibition of inflammation and fibrosis, and a significant improvement in left ventricular remodelling [164].

Cell encapsulation in hydrogels is an additional therapeutic option under investigation to reduce oxidative stress, as some cell types such as mesenchymal stem cells (MSCs) are immunomodulators [165]. A graphene oxide/laponite/gelatine (GO-LG) hydrogel was developed by Cheng et al. and loaded with mesenchymal stem cells (MSCs), showing a significant decrease in the oxidative damage in CMs [166]. In an attempt to restore infarcted myocardium, MSCs were encapsulated into a hydrazide–hyaluronic acid solution and then combined with a sponge of 2-hydroxy-*β*-cyclodextrin and resveratrol. Results showed that the sponge could protect CMs under oxidative stress in vitro and, in vivo, demonstrated an improved cardiac microenvironment and reduced CM apoptosis. Moreover, transplanted stem cells can also secrete a variety of growth factors and cytokines that promote angiogenesis, reduce cardiac fibrosis and promote cardiac repair [167].

Coating stents with antioxidant polymeric scaffolds has also been raised as a possible solution to I/R injury. Janjic et al. have developed electrospun rosuvastatin and heparin-loaded cellulose nanofibrous scaffolds as a nanocoating for vascular stents. In addition to having an antithrombotic effect, statins also have anti-inflammatory properties. They showed a controlled drug release which demonstrated potential in the development of vascular grafts with anti-thrombogenic and anti-inflammatory functions [168]. Another electrospun scaffold for stent coating with antioxidant properties has been developed by Wang et al. They have synthesized rapamycin and 4-hydroxy-2,2,6,6-tetramethylpiperidine 1-oxyl (TEMPOL)-loaded PLA/PVA electrospun membranes as ROS scavenger and vascular smooth muscle cell proliferation inhibitor. Their results in an in vivo pig model showed rapid endothelialization and ROS scavenging [169]. More studies in this area are underway and most of them work on commercially available stents, making it a very promising therapeutic approach [170,171,172].

**Figure 7:**
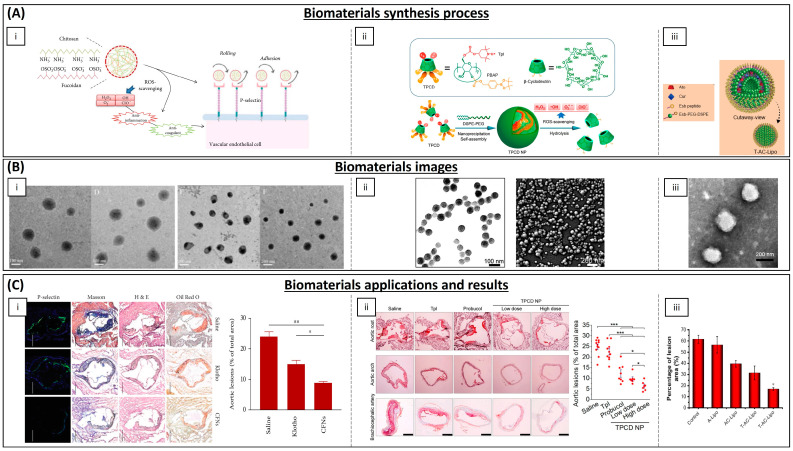
Some biomaterials currently used for the treatment of atherosclerosis. (**A**) Synthesis process of the biomaterials. (**i**) Illustration of the synthesis of CFNs with P-selectin targeting potential, to block leukocyte recruitment and rolling on platelets and endothelium in atherosclerotic plaques [185]. (**ii**) Chemical structure of a broad-spectrum ROS-scavenging material TPCD and development of a TPCD NP [186]. (**iii**) Illustration of E-selectin-targeting liposomes (T-AC-Lipo), simultaneously encapsulating atorvastatin calcium (Ato) and Cur [181]. (**B**) Biomaterials images. (**i**) TEM image of CNFs (scale bars: (**left**) 100 nm; (**right**) 200 nm) [185]. (**ii**) TEM (**left**) and SEM (**right**) images of TPCD NPs [186]. (**iii**) TEM image of T-AC-Lipo [181]. (**C**) Results of the biomaterials as treatments of neurodegenerative diseases. (**i**) These results show that targeted P-selectin delivery by CFNs strikingly attenuates atherosclerotic development and stabilizes the atherosclerotic plaques. * *p* < 0.05; ** *p* < 0.01 [185]. (**ii**) Observation on ORO-stained cryosections from the aortic sinus, aortic arch and brachiocephalic artery revealed the most significant antiatherosclerotic activity for TPCD NPs at both low and high doses. The quantitative analysis of the lesion area in aortas show that it was notably reduced after therapy with TPCD NPs. * *p* < 0.05; *** *p* < 0.001 [186]. (**iii**) Results indicate that the treatment with T-AC-Lipo resulted in a decrease in the lesion area in aortas [181]. * *p* < 0.05. All images reproduced with permission. Images (**A**(**i**),**B**(**i**),**C**(**i**)) [185] 2022, Mingying Liu et al. Images (**A**(**ii**),**B**(**ii**),**C**(**ii**)) [186] 2018, American Chemical Society. Images (**A**(**iii**),**B**(**iii**),**C**(**iii**)) [181] 2019, Li X et al.

In addition to antioxidant therapy for MI and I/R, materials with these properties have also been described for the treatment of atherosclerosis. Atherosclerosis is a progressive disease characterized by the detrimental accumulation of lipids and fibrin within arterial walls. These atherosclerotic lesions, particularly in coronary arteries, can constrict the arterial lumen and induce stenosis, leading to subsequent myocardial ischemia. Oxidation of low-density lipoproteins (LDL) by ROS activates their uptake by macrophages, leading to their accumulation and the formation of an atheroma. Thus, oxidative stress can be the leading cause of the development of atherosclerotic lesions [173]. Because ROS production has a direct impact on cell membrane components and enhances endothelial dysfunction, fibrosis, and loss of tissue structure and function can occur, providing a pathogenic environment that also promotes the development of atherosclerosis [174]. The overproduction of ROS is also influenced by the recruitment of mast cells and leukocytes, due to elevated oxygen uptake, leading to increased release and accumulation of pro-oxidant agents at the site of injury [175,176]. Likewise, the production of various cytokines and chemokines continues to attract more inflammatory cells, producing even more ROS [177]. Cytokines and inflammatory cells, together with fibrosis associated with endothelial injury, can lead to the formation of an atheroma plaque. Most of the antioxidant materials developed for atherosclerosis are NPs for drug delivery to atherosclerotic plaque sites (Table 4 and Figure 7).

Epigallocatechin gallate (EGCG)-loaded NPs with α-tocopherol and modified with the major type of oxidized phosphatidylcholines found in oxidized LDL showed in in vitro studies specific binding to macrophages. Mice treated with the NPs also had significantly smaller lesion surface areas on aortic arches compared to the controls [178]. Nanometre-sized lipid carriers loaded with EGCG and chitosan significantly augmented EGCG stability, optimized its sustained release, enhanced its cellular bioavailability, and diminished cholesterol content and chemoattractant protein expression in macrophages [179]. Curcumin has been combined with bioperine, a potent enhancer of bioavailability due to its rapid absorption characteristics, and both were loaded into PLGA NPs. Results showed downregulation of the expression of genes involved in inflammatory pathways in the atherosclerotic process in vitro [180]. Liposomes composed of cholesterol, 1,2-dipalmitoyl-sn-glycero-3-phosphocholine (DPPC) and 1,2-distearoyl-sn-glycero-3-phosphoethanolamine-*N*-(maleimide(polyethyleneglycol)2000 (Mal-PEG2000-DSPE) were loaded with atorvastatin calcium, which ameliorates atherosclerosis by lowering plasma lipid and inflammatory factors levels, and curcumin and modified using a ligand targeting dysfunctional endothelial cells. This approach effectively diminished foam cell formation, curbed inflammatory factor secretion, and lowered plasma lipid levels in in vitro and in vivo mouse models [181]. Diosmin, derived from citrus fruits, possesses antioxidant and anti-inflammatory properties, but its poor water solubility hinders its absorption through the gastrointestinal tract. To circumvent this limitation, Om et al. designed a hydroxypropyl starch and poly lactide–glycolidechitin polymeric matrix to prepare diosmin NPs. Treated rats showed significant downregulated levels of inflammatory molecules [182]. Selenium NPs stabilized with chitosan could alleviate early atherosclerotic lesions in mice after oral administration for 10 weeks accompanied by the alleviation of endothelial dysfunction and inflammation [183]. The leaf extract of *Spinacia oleracea* was used as a reducing agent to synthesize iron oxide NPs with antioxidant activity. These NPs were able to mitigate the atherosclerotic effects of Triton X-100 in vitro, with a significantly increased activity of SOD and catalase in rats [184]. Liu et al. utilized nontoxic fucoidan polysaccharide, a sulphated polysaccharide derived from the edible marine brown seaweed *Fucus vesiculosus*, to formulate chitosan–fucoidan NPs. These NPs were further modified to exhibit selective binding to P-selectin, an inflammatory adhesion molecule expressed on endothelial cells and activated platelets. This modification enabled the NPs to effectively impede leukocyte recruitment and rolling on platelets and endothelium. These NPs were shown to exhibit antioxidant and anti-inflammatory properties in vitro and effectively suppressed local oxidative stress and inflammation in mice [185]. Tempol (SOD mimetic agent) and phenylboronic acid pinacol ester (a hydrogen peroxide-eliminating compound) were encapsulated onto a cyclic polysaccharide *β*-cyclodextrin NPs. These NPs were capable of being internalized efficiently and rapidly by macrophages and vascular smooth muscle cells, effectively mitigating ROS-induced inflammation and cell apoptosis in macrophages by eliminating excessive intracellular ROS. In vivo studies in mice revealed that β-cyclodextrin NPs effectively ameliorated systemic and localized oxidative stress and inflammation, concurrently minimizing inflammatory cell infiltration within atherosclerotic plaques [186].

The development of new biomaterials such as the ones mentioned here, allowing a sustained release of antioxidants and in situ control of inflammation, has potential to become a great advance for human health, considering that cardiovascular diseases continue to be the leading cause of death worldwide. As cardiac tissue has insignificant regenerative capacity, early anti-inflammatory treatment in these diseases is essential to avoid permanent loss of damaged tissue. This makes injectable materials a better therapeutic approach due to their simpler administration. In addition, the contractility of the tissue would hinder the adhesion of an implant, in the case of polymeric scaffolds, which should also respond to this contractility. In the case of stents with antioxidant coatings, this is a very good option as modified stents containing drugs are currently used in the clinic.

**Table 3:** Antioxidant materials for I/R and cardiac infarct.

Category	Material	Load	Model	Properties	Ref.
Polymeric NPs	Copolyoxalate	Vanillyl alcohol	I/R mouse model	Reduction in ROS	[130]
	PEG and poly-(propylene sulphide)	Ginsenoside Rg3	I/R rat model	Inhibition of oxidative stress, inflammation and fibrosis	[131]
	(PLL-PEG-PLL)	-	I/R rat model	Decreased oxidative stress and promoted myocardial function	[134]
	PLGA	Quercetin	H9C2 cells	Increased quercetin bioavailability	[135]
	PLGA	Resveratrol	H9C2 cells	ROS scavenging	[136]
	PLGA	Resveratrol	Rat	Preventing myocardial necrosis	[137]
	PLGA	Pioglitazone	Mouse and porcine model	Cardioprotection	[138]
	PLGA	Irbesartan	I/R mouse model	Anti-inflammatory activity and reduced infarct size	[139]
	PLGA	Mdivi-1	I/R mouse model	Cardioprotection against I/R	[140]
	PLGA	CoQ-10	Mice	Increased bioavailability	[142]
	PGMA	AID and cur/res	Rat	Decreased oxidative stress	[145]
Solid lipid NPs	PEG-modified solid lipid NPs	Baicalin, schisandrin B	Rat	Reduction in the infarction size	[132,133]
	Egg phosphatidylcholine, cholesterol, PEG2000-DSPE	CoQ-10	I/R rabbit model	Limiting the fraction of damaged myocardium	[143]
Inorganic NPs	Ceria NPs	-	Murine cardiac progenitor cells	Protecting cardiac progenitor cells	[147]
	AuNP-MIBI	-	I/R rat model	Reduction in inflammation	[150]
Inorganic fibres	Polyurethane	Methylprednisolone	Rat	Reconstruction of cardiac function	[152]
Hydrogels	Modified chitosan (CS-B-NO)	NO	I/R mouse model	Attenuation of cardiac damage	[154]
	PMNT-PEG-PMNT	-	Mouse	ROS scavenging	[155]
	PVA/Dex	Astaxanthin	Rat	Reduction in oxidative stress	[156]
	Chitosan	α-tocopherol	Neonatal rat cardiomyocytes	Suppression of oxidative stress	[157]
	Chitosan chloride–glutathione	-	Neonatal rat cardiomyocytes	Scavenging superoxide anion and hydroxyl radical	[158]
	Chitosan–vitamin E	-	Neonatal rat cardiomyocytes	Reducing ROS	[159]
	Chitosan	Ferulic acid	Rabbit	Protection from oxidative stress	[160]
	CMC-BA	Curcumin, collagen III	Rat	Anti-inflammatory	[161]
	Alginate	Fullerenol nps	Brown adipose-derived stem cells	Scavenging the superoxide anion and hydroxyl radicals	[162]
	N-isopropyl acrylamide and methoxy-PEG methacrylate	-	Sheep	Increased contractile function and decreased ROS	[163]
	Poly(DH-SE/PEG/PPG urethane	-	Mouse	Inhibition of inflammation and fibrosis	[164]
	GO-IG	MSCs	WJ-MSCs and rat cardiomyocytes	Decreasing the oxidative damage	[166]
	Hyaluronic acid and 2-hydroxy-β-cyclodextrin	Resveratrol and MSCs	Rat	Proangiogenic, anti-inflammatory and anti-apoptotic activity	[167]
Polymeric scaffolds	Cellulose	Statin and heparin	-	Anti-thrombogenic and anti-inflammatory functions	[168]
	PLA/PVA	TEMPOL, rapamycin	Porcine model	Favours endothelialization and mitigates local inflammation	[169]

**Table 4:** Antioxidant materials for atherosclerosis treatment.

Category	Material	Load	Model	Properties	Ref.
Polymeric NPs	Chitosan	EGCG	THP-1 cells	Decreasing cholesterol content and chemoattractant protein expression in macrophages	[179]
	PLGA	Curcumin–bioperine	THP-1 cells	Anti-inflammatory activity	[180]
	Poly lactide–glycolidechitin	Diosmin	Rat	Downregulation of inflammatory molecules levels	[182]
	Chitosan	Selenium	Mice	Alleviation of early atherosclerotic lesions	[183]
	Chitosan–fucoidan	-	Mice	Suppression of local oxidative stress and inflammation	[185]
	β-cyclodextrin	Tempol, phenylboronic acid pinacol ester	Mice	Antioxidant and anti-inflammatory properties	[186]
Lipid NPs	Phosphatidylcholines	EGCG and α-tocopherol	Mice	Smaller lesion surface areas on aortic arches	[178]
	Cholesterol, DPPC and Mal-PEG_2000_-DSPE	Atorvastatin calcium and curcumin	Mice	Reduction in plasma lipid levels	[181]
Inorganic NPs	Iron oxide	*Spinacia oleracea*	Mice	Increased activity of SOD and catalase enzymes	[184]

### 4.4. Bone Diseases

Many diseases affecting the bone have been linked to oxidative stress, amongst which one of the most important is osteoporosis. It is defined as a progressive loss of bone mineral density (BMD), leading to weakened bones with reduced strength, decreased mass and impaired microarchitecture. Consequently, this condition increases the susceptibility to fractures, particularly in the spine, hip and wrist [187]. Osteoporosis affects millions of individuals worldwide, contributing to significant morbidity, mortality and economic burden [188].

Bone is a dynamic tissue that undergoes continuous renewal throughout life through the coordinated action of three main types of bone cells: osteoclasts, osteoblasts and osteocytes [189]. This process of bone remodelling is orchestrated by the intricate interactions between these cells and various molecular agents, including growth factors, hormones and cytokines. Osteoclasts remove old or damaged bone, later replaced by new tissue formed by osteoblasts. Osteocytes are considered the mechano-sensors of bone, as they have the potential to detect mechanical forces and translate them into biochemical signals [190]. Healthy bone undergoes a tightly regulated process of remodelling, ensuring minimal alterations in bone mass and mechanical strength after each remodelling cycle [191].

**Figure 8:**
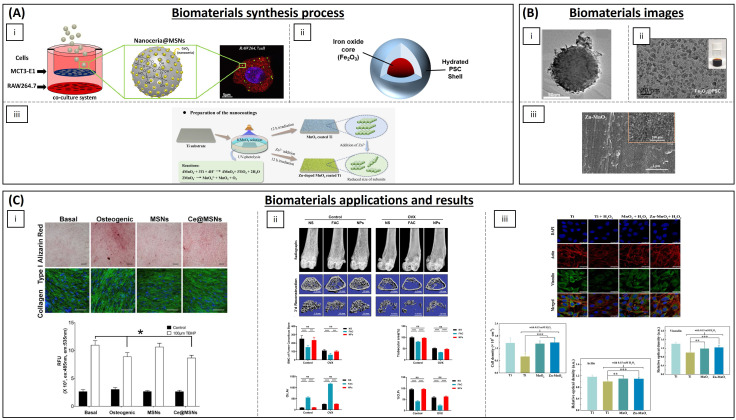
Some biomaterials currently used for the treatment of osteoporosis. (**A**) Synthesis process of the biomaterials. (**i**) Pinna A et al. have tested the capability of the ROS scavenger nanoceria encapsulated within mesoporous silica nanoparticles (Ce@MSNs) to treat osteoporosis using a co-culture system of MC3T3-E1 and RAW264.7 macrophages [202]. (**ii**) Schematic structure of Fe_2_O_3_@PSC nanoparticles (NPs) [206]. (**iii**) Preparation of the MnO_2_ and Zn-doped MnO_2_ nanocoatings via a UV-photolysis reaction. Zn^2+^ addition would inhibit the MnO_2_ crystal growth and reduce its size [216]. (**B**) Biomaterials images. (**i**) TEM image of an individual Ce@MSN [202]. (**ii**) TEM image of the NPs (scale bar: 20 nm) [206]. (**iii**) SEM image of Zn-doped MnO_2_ nanocoating [216]. (**C**) Results of the biomaterials as treatments of neurodegenerative diseases. (**i**) The effect of the Ce@MSNs on mineralization was studied using Alizarin Red staining (top row) and it was much more pronounced than in the cells cultured with MSNs and the basal condition. The green fluorescence (bottom row) show that exposure to Ce@MSNs increased expression of collagen type 1 in MC3T3-E1 cells compared to basal media and media containing MSNs. The graph indicates the Ce@MSN ability to scavenge ROS. * *p* < 0.05 [202]. (**ii**) Morphological analysis shows that the formation of osteoids increased, while the bone resorption area decreased with NP administration. Analysis of the bone mineral density of the femur cancellous bone (**top left**), trabecular area (**top right**), area of osteoclasts (Oc. Ar) (**bottom left**) and percentage of osteoid perimeter (%O. Pm) (**bottom right**) shows significant differences between FAC (ferric ammonium citrate) treatment and NP administration. *p* > 0.05; *** *p* < 0.001; **** *p* < 0.0001. [206]. (**iii**) Images show that H_2_O_2_ treatment suppressed the recruitment of cellular actin and vinculin in cells for the Ti + H_2_O_2_ group. In contrast, the enhanced actin microfilaments and vinculin dots were observed in H_2_O_2_-treated cells on the MnO_2_ and Zn-MnO_2_ nanocoatings. Quantitative analysis (graphs) revealed the same results. ** *p* < 0.01; *** *p* < 0.001 [216]. Scale bar: 25 μm. All images reproduced with permission. Images (**A**(**i**),**B**(**i**),**C**(**i**)) [202] 2021, Elsevier. Images (**A**(**ii**),**B**(**ii**),**C**(**ii**)) [206] 2020, Elsevier. Images (**A**(**iii**),**B**(**iii**),**C**(**iii**)) [216] 2021, Elsevier.

The pathophysiology of osteoporosis has been related to oxidative stress. Repeated stressful loading events promote the development of submicron fractures. The remodelling process of these small fractures induces the formation of new capillaries, augmenting the vascularization of the site and stimulating the influx of inflammatory cells, leading to a subsequent rise in ROS [192]. Excessive ROS levels can detrimentally impact osteoblast lifespan and ECM deposition [193,194]. Moreover, ROS can impede and diminish the activity and differentiation of osteoblasts. In addition, they also favour their apoptosis as well as the apoptosis of osteocytes, hindering mineralization and osteogenesis [195,196]. On the other hand, oxidative stress activates the differentiation of preosteoclasts into osteoclasts reinforcing bone resorption [197]. These events cause an imbalance between the activity of osteoblasts and osteoclasts resulting in a decrease in bone mineral density leading to osteoporosis [198] and suggesting that oxidative stress could be the main inducer of the bone deterioration effect. In addition, oxidative stress has been linked to activation of NADPH oxidase and decreased synthesis of antioxidant enzymes in bone due to hormonal regulation [199,200]. Therefore, biomaterials with antioxidant and anti-inflammatory activity for the treatment of osteoporotic bone defects are a promising option. They can be mainly divided into hydrogels, polymeric NPs and inorganic NPs (Table 5 and Figure 8).

Silibinin, a polyphenolic flavonoid active compound extracted from the seeds of *Silybum marianum*, was combined with selenium hydrogel scaffolds by Tao et al., demonstrating a potent effect on bone regeneration and bone mineralization, and enhanced expression of antioxidant and osteogenic proteins in vitro and in vivo in an osteoporotic rat model [201].

Referring to polymeric NPs, nanoceria has also been employed for osteoporosis treatment due to its known antioxidant activity. Nanoceria encapsulated within mesoporous silica NPs demonstrated antioxidant potential, effectively stimulated cell proliferation and promoted osteogenic responses without the need for additional osteogenic supplements in vitro [202]. Li et al. successfully synthesized a plasma-sprayed CeO2 coating with a hierarchical topography, featuring a micro-rough surface onto which ceria NPs were effectively superimposed. This approach showed reduced SOD activity and decreased reactive oxygen species production in H_2_O_2_-treated osteoblasts [203]. Shilajit, a natural mineral substance with multiple components, has demonstrated effectiveness in modulating immunity, exhibiting antioxidant activity and promoting disease resistance [204]. Nanochitosan conjugation with shilajit water extract could cause anti-osteoporotic activity by reducing oxidative stress, cytokines and H_2_O_2_ while restoring antioxidant levels in rats [205]. The biological polysaccharide-based antioxidant polyglucose-sorbitol-carboxymethyl ether (PSC) was used by Yu et al. as the precursor to synthesize Fe_2_O_3_@PSC NPs. These NPs could scavenge ROS, promoted osteogenic differentiation and inhibited osteoclast differentiation in vitro and in vivo [206]. Tocotrienol, a member of vitamin E family, possesses antioxidant capacity. To increase its bioavailability, Gao et al. have combined it with PLGA NPs and injected into the bones of a postmenopausal osteoporosis rat model, achieving higher mineral content and an improvement in bone strength, compared with controls [207]. Polaprezinc (Zinc *L*-carnosine), a commercially available drug that acts as a potent inducer of antioxidant enzymes such as SOD and glutathione peroxidase, was effectively incorporated into polycaprolactone/gelatine hybrid electrospun nanofibres to produce a membrane with antioxidant and pro-osteogenesis capabilities. Scaffolds were implanted into the cranial bone defects of osteoporotic rats. The results showed that the scaffolds were able to decrease oxidative stress and enhance bone formation [208].

Regarding inorganic NPs, selenium has also been employed. Selenium NPs have demonstrated the capacity to preserve mechanical and microstructural properties of bone in vivo. Fatima et al. showed that selenium NPs increased antioxidant levels in human MSCs undergoing osteogenesis compared to untreated cells [209,210]. Lycopene is a carotenoid and antioxidant molecule that has been employed to form NPs by Ardawi et al. resulting in an enhanced osteoblast differentiation from MSCs [211]. Zheng et al. have demonstrated that daily administration of iron oxide NPs promoted the osteogenic differentiation of bone marrow MSCs and inhibited the osteoclast differentiation of monocytes in mice via scavenging ROS [212]. Platinum NPs exhibit great antioxidant activity. They were administered intragastrically in mice, achieving a decrease in the level of activity and number of osteoclasts, as well as ROS and oscillation in intracellular Ca^2+^ concentrations [213]. Mn-containing *β*-tricalcium exhibited superior scavenge capability against oxygen and nitrogen radicals, suggesting its antioxidant properties compared to *β*-tricalcium alone. In vitro and in vivo studies demonstrated that released Mn^2+^ ions effectively inhibited osteoclast formation and activity, enhanced osteoblast differentiation, and accelerated bone regeneration under osteoporotic conditions in rats [214].

On the other hand, when an osteoporotic fracture occurs, the use of orthopaedic implants is necessary. However, osseointegration in osteoporosis after prosthesis implantation is often inadequate, leading to an increased risk of complications, such as prosthesis displacement and loosening, as well as periprosthetic fractures. This is primarily attributed to the progression of the disease, which is accompanied by substantial inflammation and ROS production [215]. To enhance prosthesis implantation, a Zn^2+^-doped MnO_2_ nanocoating was readily incorporated onto orthopaedic titanium implants via a UV-photolysis reaction. This nanocoating exhibited catalase-like activity and effectively suppressed intracellular oxidative product generation, shielding SOD levels from depletion under H_2_O_2_-induced oxidative stress. This, in turn, safeguarded pre-osteoblast functions in vitro [216]. Riccucci et al. have developed a hydroxyapatite-based material with a coating of chitosan grafted with polyphenols which enables a controlled delivery of the antioxidant molecules [217]. Large titanium (TiO_2_) nanotubes exhibited substantially enhanced osteogenic differentiation and demonstrated superior cellular outcomes in vitro, featuring augmented osteoblast adhesion, survival and differentiation compared to native titanium (control). Consequently, these nanotubes could be advantageous for the development of orthopaedic implants for patients with bone degenerative diseases [218]. Ding et al. developed a ROS-scavenging hydrogel by crosslinking EGCG, 3-acrylamido phenylboronic acid and acrylamide, and encapsulated bone marrow MSCs within it. The hydrogel effectively enhanced osteointegration of 3D-printed microporous titanium alloy prostheses in an osteoporotic rabbit model, exhibiting the ability to scavenge accumulated ROS, suppress inflammatory cytokine expression and improve osteogenesis-associated markers [219]. Melatonin has been shown to possess free-radical scavenging and antioxidant properties, complementing its crucial role in bone formation [220]. Xiao et al. have constructed a composite adhesive GelMA-dopamine hydrogel to bring about sustained melatonin release that could inhibit osteoblast apoptosis caused by oxidative stress. This in turn promoted osteogenesis and improved bone quality around titanium implants inserted into rat bones [221].

In the case of NPs, they are presented as a solution to the development of osteoporotic disease by preventing a possible fracture. Injectability and easy administration make this type of biomaterial a good therapeutic strategy for cases of early osteoporosis. However, once the fracture has occurred, it would be necessary to resort to traditional hard materials to replicate the mechanical properties of bone; complementing these materials by formulating them with antioxidant properties or adding an antioxidant coating may allow better integration of the prosthesis and prevent the progression of the disease.

As a whole, these recent investigations have demonstrated that a diverse range of bone scaffolding materials and functionalized molecules with antioxidant properties have the potential to enhance patient clinical outcomes. These findings suggest that interdisciplinary research is leading to the development of novel technologies for regenerative medicine, oxidative stress management, bone disease prevention and osteogenesis promotion.

**Table 5:** Antioxidant materials for osteoporosis treatment.

Category	Material	Load	Model	Properties	Ref.
Hydrogel	Poloxamer 407 and selenium	Silibinin	Rat	Bone regeneration and mineralization	[201]
	EGCG, 3-acrylamido phenylboronic acid and acrylamide	MSCs	Rabbit	Antioxidant and anti-inflammatory activity, and improved osteogenesis	[220]
	gelatine methacryloyl–dopamine	Melatonin	Rat	Promotion of osteogenesis and improved bone quality	[221]
Polymers	Silica NPs	Cerium oxide	RAW264.7 and MC3T3-E1 cells	Antioxidant capability and stimulated cell proliferation and osteogenic responses	[202]
	Chitosan NPs	Shilajit water extract	Rat	Antioxidant and anti-inflammatory activity	[205]
	Fe_2_O_3_@PSC NPs	-	Mice	ROS scavenging, pro-osteogenic and inhibition of osteoclast differentiation	[206]
	Lycopene NPs	-	BMSCs	Pro-osteoblast differentiation	[211]
	PLGA NPs	Tocotrienol	Rat	Improvement in bone strength and mineralization	[207]
	polycaprolactone/gelatine NPs	Polaprezinc	Rat	Promotion of bone formation	[208]
	Titanium dioxide nanotubes	-	Rat calvarial osteoblasts	Improvement in osteoblast adhesion and osteogenic differentiation	[218]
Inorganic NPs	Selenium	-	hESC-derived hMSCs	Increased antioxidant levels	[209]
	Cerium oxide	-	MC3T3-E1 cells	Antioxidant activity	[203]
	Iron oxide	-	Mice	Antioxidant, osteogenic differentiation and inhibition of osteoclast differentiation	[212]
	Platinum	-	Mice	Decreased osteoclast activity levels and antioxidant capacity	[213]
	Manganese	β-tricalcium	Rat	Promotion of the differentiation of osteoblasts and accelerate bone regeneration	[214]
	Manganese oxide	Zn^2+^	MC3T3-E1 cells	Catalase-like activity	[216]

## 5. Discussion and Future Perspectives

The use of materials- and TE-based approaches to address the pathological effects of oxidative stress has seen explosive research in the last decade. Given the medical, economic and humane impact of targeted diseases, such as the ones reviewed here, these applications are poised to make significant contributions to their treatment, as well as their understanding. However, if the field is to progress beyond the present plethora of preclinical studies, there are a number of challenges that must be addressed. The first is standardization. As depicted in the sections above, many antioxidants rely on natural products or extracts. These, although they can be highly effective, are often not fully defined in their characterization or methodology, increasing the risk of batch-to-batch variations and lack of reproducibility. Therefore, improved and more detailed methods must be developed and disseminated. Secondly, the field, as many others, is highly impacted by a lack of a translational thinking and progress. Many of the studied compounds or formulations, albeit their benefits, are not pursued further towards the bedside. One of the reasons is the enormous gap between a naturally derived molecule and a clinically approved product, where modifications in processes can result in a lack of effect for the refined compound. Also, the use of under-representative animal models can hinder the progressing to the clinical stage. Here, use of more relevant testbeds, such as those based on human induced pluripotent stem cell (hiPSC)-derived phenotypes, bioengineered technologies (e.g., organ-on-a-chip) and advanced AI-supported computational models, can provide a more reliable bridging between preclinical and clinical stages, as recently promoted by the FDA [222,223,224]. Thirdly, issues such as dosing, time of delivery and route of administration will have to be brought into play and pinned down, in order to achieve a maximized therapeutic effect for a given application. Here, again, species-specific differences can pose an important hindrance.

In conclusion, the use of biomaterials with the capacity to limit oxidative stress or to incorporate molecules with this ability is poised to produce a significant impact on human health. Thus, we can presume that the development of such interdisciplinary studies will lead to the emergence of new technologies for TE, control of oxidative stress and prevention of diseases associated with oxidative stress in the following decade.

## Data Availability

No new data were created or analyzed in this study. Data sharing is not applicable to this article.
